# Selective mutism and the risk of mental and neurodevelopmental disorders among siblings

**DOI:** 10.1007/s00787-022-02114-3

**Published:** 2022-11-23

**Authors:** Miina Koskela, Elina Jokiranta-Olkoniemi, Terhi Luntamo, Auli Suominen, Andre Sourander, Hans-Christoph Steinhausen

**Affiliations:** 1 Department of Child Psychiatry, University of Turku, Turku, Finland; 2 Department of Child Psychiatry, Turku University Hospital, Turku, Finland; 3 INVEST Research Flagship Center, University of Turku, Turku, Finland; 4 Unit of Psychology, Faculty of Education, University of Oulu, Oulu, Finland; 5 Department of Child and Adolescent Psychiatry, Psychiatric University Clinic, Zurich, Switzerland; 6 Clinical Psychology and Epidemiology, Department of Psychology, University of Basel, Basel, Switzerland; 7 Department of Child and Adolescent Psychiatry, University of Southern Denmark, Odense, Denmark; 8 Child and Adolescent Mental Health Centre, Capital Region Psychiatry, Copenhagen, Denmark; 9 Research Centre for Child Psychiatry, Faculty of Medicine, University of Turku, Turku, Finland

**Keywords:** Selective mutism, Family clustering, Epidemiology, Mental disorders, Sibling study

## Abstract

The siblings of children with mental disorders are more likely to experience mental health issues themselves, but there has been a lack of sibling studies on selective mutism (SM). The aim of this population-based study was to use national registers to examine associations between children with SM and diagnoses of various mental disorder in their siblings. All singleton children born in Finland from 1987 to 2009, and diagnosed with SM from 1998 to 2012, were identified from national health registers and matched with four controls by age and sex. Their biological siblings and parents were identified using national registries and the diagnostic information on the siblings of the subjects and controls was obtained. The final analyses comprised 658 children with SM and their 1661 siblings and 2092 controls with 4120 siblings. The analyses were conducted using generalized estimating equations. Mental disorders were more common among the siblings of the children with SM than among the siblings of the controls. The strongest associations were observed for childhood emotional disorders and autism spectrum disorders after the data were adjusted for covariates and comorbid diagnoses among SM subjects. The final model showed associations between SM and a wide range of disorders in siblings, with strongest associations with disorders that usually have their onset during childhood. Our finding showed that SM clustered with other mental disorders in siblings and this requires further research, especially the association between SM and autism spectrum disorders. Strong associations with childhood onset disorders may indicate shared etiologies.

**Supplementary Information:**

The online version contains supplementary material available at 10.1007/s00787-022-02114-3.

## Introduction

Selective mutism (SM) is characterized by the inability to speak in certain situations, despite being able to speak in others. It is now considered an anxiety disorder [1]. Symptoms appear consistently in certain social situations, for example not being able to speak at school, but speaking normally at home. The International Classification of Diseases, Tenth Revision (ICD-10), states that selective mutism can be diagnosed if the symptoms last for at least one month, excluding the first four weeks of school. SM cannot be diagnosed with autism spectrum disorder (ASD) or psychotic disorders [2]. In addition, The Diagnostic and Statistical Manual of Mental Disorders, Fifth Edition (DSM-V), states that the disturbance caused by SM must interfere with educational achievements or social communication, is not explained by a communication disorder and does not occur exclusively during the course of ASD or psychotic disorders [3]. The prevalence of SM tends to be relatively low, varying from 0.18 to 1.9% in published studies, depending on the study protocol and diagnostic criteria used [4, 5]. Follow-up studies have reported that the symptoms of SM lasted a long time, but improved during follow-up, and that social phobias, phobic disorders and communication problems were common later in life [6–8]. Although academic skills and abilities did not seem to differ from average levels in some studies [9, 10], difficulties in communication might have affected academic performance [5].

Given the limited understanding of the etiology of SM, there are three specific research areas that are of particular interest when examining the risk of psychopathology transmission in these individuals. These are (1) family studies of mental disorders and SM, (2) sibling studies of mental disorders and SM and (3) studies of mental comorbidities and SM. Addressing these subjects could help us to discover if SM runs in families and whether it shares environmental or genetic risk factors with other disorders.

### Family studies of mental disorders and SM

Family studies that have examined the associations between children with SM and mental disorders among family members have mainly investigated parental diagnoses [11–15]. These studies have shown increased levels of parental mental disorders among children with SM and the findings were not just restricted to parental anxiety disorders [11–13, 16]. A recent register-based study on SM and parental mental disorders by the present authors found higher rates of several kinds of disorders among the parents of the subjects than among the parents of the controls. These included psychotic disorders, mood disorders and personality disorders [11]. Similarly, non-specific family clustering, among family members, has been reported for a number of other mental disorders [17–20]. However, the findings of family studies have not been completely consistent, as two studies only found associations between paternal, but not maternal, psychiatric morbidity and offspring SM [14, 15].

### Sibling studies of mental disorders and SM

In contrary to studies on parental mental disorders and SM, there have only been a few studies that have examined mental disorders among the siblings of children with SM. These studies have focused on mental disorders as a single variable or have only focused on SM diagnoses among the siblings of subjects. Most of them did not have control groups. A study that carried out interviews with the parents of children diagnosed with SM reported that 16% of the children’s siblings had mental disorders. The rate was only 4.9% in the control group siblings in that study [21]. In contrast, another case–control-study, which was based on interviews with parents, found no associations between children with a clinical diagnosis of SM and a history of mental disorders in their siblings [22]. One school-based sample used parental questionnaires and DSM-III criteria and reported that the prevalence of SM among siblings was almost 20% [23]. Meanwhile, a study that used a clinical sample of children diagnosed with SM found that about 18% of their siblings also displayed mutistic reactions. However, it did not specifically address SM diagnoses among the siblings. [7]

### Studies of mental comorbidities and SM

Comorbidities of SM have been addressed quite well in recent literature and SM has been associated with several comorbid diagnoses. A meta-analysis found that up to 80% of subjects with SM had some kind of comorbid anxiety disorder [24] and the most common diagnosis was social phobias (69%). The associations between SM and neurodevelopmental disorders have been investigated by a few studies [21, 25, 26] and these showed that 30–40% of subjects with SM also had learning and coordination disorders [27, 28]. The relationship between SM and ASD has also been discussed [29]. One study found that as many as 63% of subjects with SM also fulfilled the diagnostic criteria for ASD. However, the diagnoses were retrospectively assessed from medical records and only included subjects from a neurodevelopment clinic. This means that the results cannot be generalized to the wider population [30].

### Aim

The aim of the present study was to use nationwide population-based data to investigate wide-ranging mental and neurodevelopmental morbidities that may affect the siblings of children with SM. To the best of our knowledge, there have not been any previous population-based studies that have investigated mental disorders among the siblings of subjects with SM. Sibling studies on SM are needed to extend our knowledge on its etiological background. Furthermore, studies that analyze the whole spectrum of diagnoses among siblings provide a starting point for researchers who aim to study family clustering between the various disorders. Previous studies conducted with the parents of children with SM have shown non-specific patterns of various mental disorders [11–13, 16]. We hypothesized that the rate of various disorders would also be increased among the siblings of children with SM.

## Methods

### Data

The data used in the present study were based on the Finnish Prenatal Study of Anxiety disorders (FIPS-Anx), which is an epidemiological study that uses a nationwide population-based sample [31]. The FIPS-Anx uses register-based information to examine the association between early developmental factors and family histories of various anxiety disorders.

The cohort for the current study comprised all singleton children born in Finland between 1 January 1987 and 30 March 2009, who were diagnosed with SM between 1 January 1998 and 31 December 2012. The full siblings were born between 1 January 1977 and 31 December 2012, and diagnoses given after 1 January 1996 were included. Subjects, controls and their respective siblings and parents were observed until 31 December 2016 and all mental disorder diagnoses that were registered during the observation years were collected. ICD-10 was in use in Finland at the time of the study. The start date of 1998 was chosen because that was the earliest date when both inpatient and outpatient data were available in the relevant registers.

The data were collected from three nationwide Finnish registers: The Finnish Hospital Discharge Register (Discharge Register), The Finnish Central Population Register (Population Register) and The Finnish Medical Birth Register (Birth Register).

The Discharge Register was established in 1969 and includes information on all mental disorders diagnosed delivered by specialized health care services. It covers all public specialized health care inpatient care units since 1969 and all outpatient units since 1998. This means that all the diagnoses delivered to subjects included in the present study during the observation years were obtained. These comprised the subjects with SM and the controls and their siblings and parents. In Finland, all diagnoses were based on ICD-9 [32] from 1987 to 1995 and on ICD-10 [2] from 1996 onwards.

The Birth Register provides data on children with SM, and their mothers, during pregnancy and delivery. The Register includes information on all live births in Finland since 1987 and the coverage is estimated to be 100% [33]. It was also used to identify controls, and their mothers, and to obtain the data on covariates, including maternal socioeconomic status (SES) and maternal age and marital status during the delivery.

The Population Register is maintained by the Finnish Population Center and local register offices. It contains personal information on all Finnish citizens, such as their name, family members and address, and was used to identify the fathers and full siblings of the subjects and controls. Information on paternal age was also obtained from this Register.

The data collected from the different registers were linked using the personal identity codes that are issued to all Finnish residents at birth or when they become permanent citizens after immigration. The study was approved by the Ethical Committee of the Hospital District of Southwest Finland.

### Subjects and their controls

All the children with SM were registered with the ICD-10 code F94.0 and matched with four controls by their birth date (± 30 days) and sex. The study included subjects who were diagnosed at least once from 3 to 15 years of age. The exclusion criteria for the subjects and controls were coexisting ASD (ICD-10 F84.0-0.9, ICD-9 229), psychotic disorders (ICD-10 F20-25, F28-29, ICD-9 295, 297, 2989X, 3012C) and a moderate or severe intellectual disability (ICD-10 F72-73, ICD-9: 318). The data used in the present study were derived from the larger FIPS-Anx sample, which had excluded controls with anxiety and childhood emotional disorders when the data were originally extracted from the national registers (ICD-10 codes F40–42, F43.0, F43.1, F43.22, F43.23 and F93-94). These categories do not include depressive disorders. This procedure ensured that the controls did not have any of the disorders that were being studied. However, some controls were subsequently diagnosed with these conditions during the observation period that followed the original data extraction. If this happened, they were excluded in line with the original criteria [31].

### Identifying the siblings

**Fig. 1:**
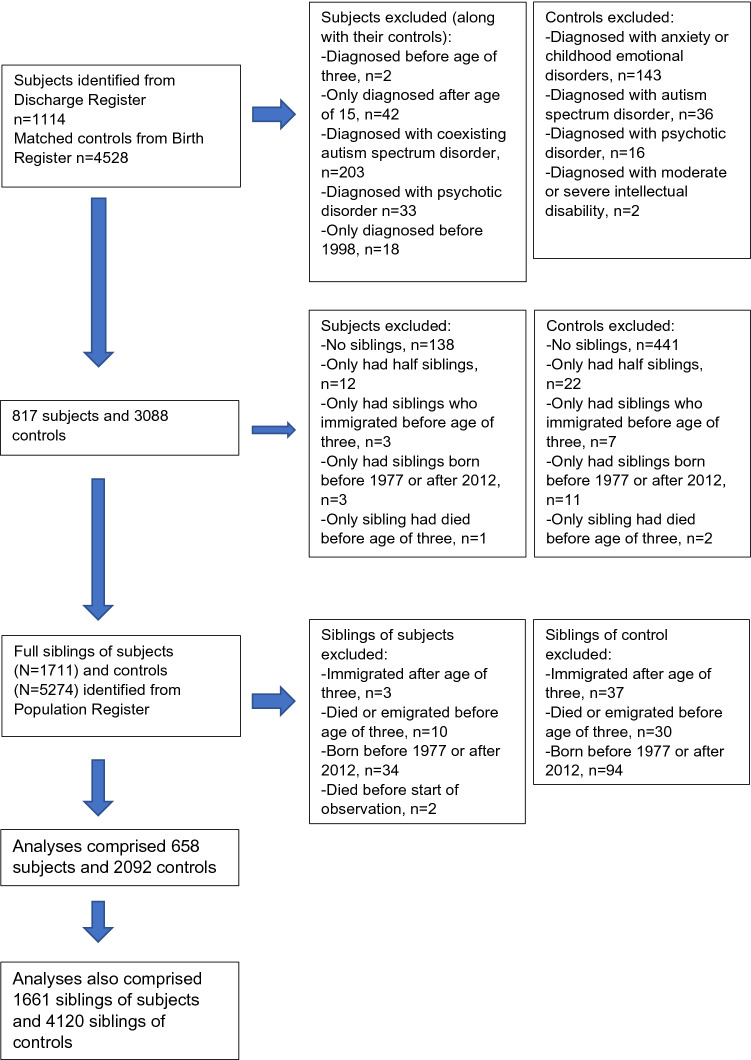
Selection of the study population

Full siblings of the subjects with SM and the controls were identified from the Population Register. A flow chart of the sample selection is shown in Fig. 1. This shows that the siblings were born between 1 January 1977 and 31 December 2012 and mental disorder and neurodevelopmental diagnoses from 1 January 1996 to 31 December 2016 were included. Siblings born before 1977 or after 2012 were excluded to ensure that there had been sufficient observation time when ICD-10 was used in Finland. Siblings who had died or emigrated before the age of three, or had moved to Finland after age of three, were excluded. Complete matched sets of subjects and controls were included if the subject, and at least one control, had one full sibling or more. We did not exclude siblings who had been diagnosed with SM because we were also interested in examining SM among the siblings, as these studies are scarce. The significance of this dependence was assumed to be low and, to control for this, the unit in each of the analyses was the sibling. A similar approach has also been used in other sibling studies, including those on subjects with attention deficit hyperactivity disorder (ADHD) and ASD [18, 19]. Thus, a total of 658 children with SM with 1697 siblings and 2092 controls with 4211 siblings were included in the final analyses.

### Diagnoses among siblings

First, we established if any of the siblings in the SM and control groups had mental disorders and this was scored as a yes if there was at least one sibling diagnosed with a mental or neurodevelopmental disorder. These siblings were then assigned to the main diagnostic categories based on their ICD-10 diagnoses (Supplementary Table 1). Due to strong associations between SM and anxiety disorders, various anxiety diagnoses were analyzed separately (Supplementary Table 2). If a sibling had received some of the diagnoses in a category at least once during the observation period, they were assigned to that category. If there were co-occurring disorders, the subject was separately assigned to each category. If the diagnosis was given to the sibling multiple times, only the first diagnosis was considered. This means that we have reported the incidence rates, not the number of diagnoses.

### Covariates

A series of potential covariates were included in the analyses, based on the findings of various studies, namely, maternal SES [11, 34], maternal mental disorder diagnoses [11, 12, 35], paternal mental disorder diagnoses [14, 15, 36], maternal and paternal age [37] and maternal marital status [11, 38, 39]. Maternal SES was divided into four categories, based on occupational class, namely upper white collar, lower white collar, blue collar and other [40]. Maternal SES has been shown to be a strong indicator of health inequality in Finland [41]. Maternal SES was only registered for subjects born from 1991 onwards, as it had not been recorded before that point. Maternal and paternal mental disorder diagnoses were used as a variable to describe how many different mental disorder diagnoses parents had received during the observation period. Parental age was used as a continuous variable. Complete data were available for maternal and paternal mental disorder diagnoses and age. Maternal marital status was classified as single or as married or cohabiting. Marital status and maternal SES are provided direct to the register and this means that missing information, due to lack of reporting, was possible.

Maternal SES, maternal marital status and parental age were all obtained during the birth of the subject. Parental mental disorder diagnoses were collected from 1 January 1987 until the end of observation period on 31 December 2016.

### Statistical analyses

The number of siblings that the subjects and controls had were compared with Fisher’s exact test, using a Monte Carlo simulation approach. Any interaction between the sex of the subjects and the outcome was tested using the Joint test. Regression models were used to estimate the associations between subjects with SM and siblings with mental and neurodevelopmental disorders. Associations between exposure and outcomes, namely selective mutism in subjects and mental disorder categories in siblings, were estimated separately. The unit of analysis was the siblings. Generalized estimating equations (GEE) for logistic regression models were used to account for matching between the subjects and controls and their siblings. Each stratum included the siblings of one child with SM and the siblings of their matched controls. Observation years were used as an offset in all models, as siblings were observed for different lengths of time. The associations between children with SM and the outcomes in their siblings are reported as odds ratios (ORs) with 95% confidence intervals (CIs). P*-*values were calculated using Pearson’s chi-square test and values of less than 0.05 were considered statistically significant. The statistical analyses were performed with SAS software version 9.4 (SAS Institute Inc, Cary, NC, USA).

Associations between mental disorders in the siblings and covariates were tested with Pearson’s chi-square test and those with P-values of less than 0.1 were added as covariates to Model 2, which was conducted for coherent data. In the final model, namely Model 3, comorbid diagnoses among subjects with SM were used as covariates, along with the previously used covariates. Comorbidity was used as a dichotomous variable (yes/no) and the subjects with SM were placed in the yes category if they had received any comorbid diagnosis at least once.

This means that there were three models conducted. Model 1 was unadjusted. Model 2 was based on Model 1 and adjusted for significant covariates, which were chosen based on the covariate analyses. In the final model, Model 3, the results were adjusted by the comorbid diagnoses of the subjects with SM and by the covariates chosen based on the covariate analyses.

In addition, we conducted sensitivity analyses on siblings in the SM group who were born between 1 January 1995 and 31 December 2009, to see whether possible changes in the methods of registration during observation years affected the results. These included adding outpatient diagnoses and possible changes in clinical practice due to increased knowledge about SM. Unadjusted analyses were conducted on this subgroup using GEE and the results were compared to the complete data to check whether the same diagnostic categories remained significant.

In addition, sensitivity analyses were conducted for the subgroup of siblings of the subjects with SM that had not been diagnosed with anxiety disorders or childhood emotional disorders. These were then compared to the siblings of their matched controls. These analyses were conducted to examine whether the results were representative, due to excluding these disorders among the controls. Similarly, unadjusted analyses were conducted, and the results were compared to the results from the complete data.

Subjects with ASD, psychotic disorders or moderate or severe mental disability were excluded, because they could not be reliably diagnosed with SM according to ICD-10. However, to explore if there were selective drop-outs due to this exclusion, the characteristics of this excluded group were compared to the complete data. P-values were calculated using Pearson’s chi-square test.

## Results

The descriptive information on the siblings is presented in Table 1. The data comprised 658 subjects with SM (59.4% girls) with 1661 siblings and 2092 controls (59.2% girls) with 4120 siblings. On average, the children diagnosed with SM had more siblings than the controls, at 2.5 versus 2.0 (*P* < 0.0001) (Table 1).

**Table 1:** Descriptive information on the siblings of children with SM and controls

	Subjects (*N* = 658)	Controls (*N* = 2092)
Number of siblings		
1	231 (35.1%)	1055 (50.4%)
2	224 (34.0%)	659 (31.5%)
3 or more	203 (30.9%)	378 (18.1%)
Mean	2.5	2.0
Range	1–17	1–14
Median (IQR)	2 (1–3)	1 (1–2)
Age when siblings first diagnosed*		
Mean (SD)	11.3 (6.3)	13.5 (6.9)
Range	0.3–34.0	0.2–33.7
Age at the end of follow-up**		
Mean (SD)	15.6 (7.3)	15.3 (7.5)
Range	0.3–35.7	0.008–35.9
Sex of siblings		
Female *n* (%)	812 (48.9)	2015 (48.93
Male *n* (%)	849 (51.1)	2105 (51.1)
Sex of subjects/controls		
Female *n* (%)	391 (59.4)	1239 (24.8)
Male *n* (%)	267 (40.6)	853 (24.1)
Age differences in families***		
Mean (SD)	− 0.15 (5.7)	− 0.4 (5.8)
Range	− 25.2–16.6	− 21.4–20.6

*SD * standard deviation

*Age of the subject with SM or the control, when their siblings received first mental disorder diagnosis

**Until end of observation period, death or emigration

***Compared to subject/control

Covariate analyses are presented in Supplementary Table 3. Only selected maternal variables showed significant associations with both SM in the subjects and the outcome of any mental disorder diagnoses. These variables, which were maternal SES, marital status and maternal mental disorder diagnoses, were included as covariates in the adjusted models. The paternal variables or maternal age did not have significant associations with both the exposure and outcome and they were not added as covariates. Maternal SES was missing for 14.3% of the subjects and 15.4% of the controls. This included 7.8% and 7.7%, respectively, who were born before 1991, when these data were not recorded.

The mental disorder diagnoses for all the siblings are presented in Table 2. The results show that 271 (41.2%) of the subjects with SM and 435 (20.8%) of the controls had a sibling diagnosed with any mental or neurodevelopmental disorder during the observation period. In the unadjusted model, Model 1, the risk for a sibling having any mental disorder diagnosis or any childhood onset diagnosis was significantly higher for the children with SM than for the controls. The highest ORs were observed for childhood emotional disorders and ASD. All the same diagnostic categories remained significant in Model 2, after adjusting for the covariates, but relatively higher risk ratios were observed in the unadjusted model.

**Table 2:** Associations between SM and mental and neurodevelopmental disorders among siblings of children with SM and matched controls

Sibling diagnosis	Subjects (*N* = 658) *n* (%)	Controls (*N* = 2092) *n* (%)	Model 1 unadjusted OR (95% CI)	Model 2^a^ adjusted OR (95% CI)	Model 3^b^ adjusted OR (95% CI)
Any mental or neurodevelopmental disorder	271 (41.2)	435 (20.8)	1.7 (1.5–1.9)***	1.6 (1.4–1.9)***	1.5 (1.2–1.8)***
Schizophrenia spectrum disorders	16 (2.4)	25 (1.2)	1.5 (0.8–2.7)	1.5 (0.7–2.9)	1.3 (0.7–2.7)
Affective disorders	100 (15.2)	165 (7.9)	1.5 (1.2–1.9)**	1.5 (1.1–1.9)*	1.4 (1.0–1.9)*
Bipolar disorders	2 (0.3)	16 (0.8)	0.3 (0.1–1.3)	N/A	N/A
Unipolar disorders	99 (15.1)	158 (7.6)	1.6 (1.2–2.0)**	1.5 (1.2–2.0)*	1.5 (1.1–2.0)*
Anxiety disorders	76 (11.6)	110 (5.3)	1.8 (1.4–2.5)***	1.6 (1.1–2.2)*	1.6 (1.1–2.3)*
Other neurotic and personality disorders	74 (11.3)	159 (7.6)	1.2 (0.9–1.5)	1.1 (0.9–1.5)	1.04 (0.8–1.5)
Substance abuse disorders	18 (2.7)	42 (2.0)	1.1 (0.7–1.8)	0.9 (0.5–1.6)	0.9 (0.5–1.8)
Any childhood onset disorder	198 (30.1)	222 (10.6)	2.3 (1.9–2.8)***	2.3 (1.8–2.8)***	1.9 (1.5–2.5)***
Autism spectrum disorders	23 (3.5)	20 (1.0)	2.6 (1.4–4.8)*	3.1 (1.6–6.0)**	2.9 (1.3–6.6)*
Attention deficit hyperactivity disorder	38 (5.8)	41 (2.0)	2.3 (1.5–3.5)**	2.8 (1.7–4.7)***	2.3 (1.2–4.5)*
Intellectual disability	12 (1.8)	19 (0.9)	1.6 (0.8–3.5)	1.5 (0.6–3.4)	1.5 (0.5–4.3)
Childhood emotional disorders	95 (14.4)	39 (1.9)	6.1 (4.2–8.9)***	5.0 (3.3–7.6)***	4.7 (2.9–7.7)***
Conduct and oppositional disorder	35 (5.3)	40 (1.9)	2.2 (1.4–3.5)**	2.9 (1.7–4.9)***	2.2 (1.0–4.4)*
Tic disorders	7 (1.1)	14 (0.7)	1.4 (0.5–3.6)	1.7 (0.6–4.8)	1.5 (0.7–3.2)
Learning and coordination disorders	104 (15.8)	136 (6.5)	1.9 (1.5–2.4)***	1.8 (1.4–2.4)***	1.4 (1.0–1.9)*

*< 0.05 **: < 0.001 ***: < 0.0001

^a^Model 2: adjusted for maternal mental disorder history, maternal socioeconomic status and marital status

^b^Model 3 (final model): adjusted for all covariates used in Model I and comorbidities of the subject with SM

The results in the final model, Model 3, were adjusted for the covariates and comorbid diagnoses of the subjects with SM. This showed significant associations between SM and any mental or neurodevelopmental disorders and any childhood onset disorders. When we examined the specific disorder categories, the highest OR was observed for childhood emotional disorders, followed by ASD, ADHD, conduct and oppositional disorders, anxiety disorders, unipolar disorders and learning and coordination disorders. There was no interaction between sibling mental disorder diagnoses and the sex of the child with SM (*P* = 0.1299).

Table 3 presents the separate sibling analyses for various anxiety categories. As shown in the table, statistically significant findings were observed for SM, generalized anxiety, social phobias and non-specific anxiety disorders, particularly in the final model. The highest ORs were seen for SM among the siblings, as 7.3% of the subjects had a sibling with SM, compared with 0.1% for the siblings of the controls. No significant associations were observed for panic disorder, separation anxiety disorder and specific phobias.

**Table 3:** Associations between SM and various anxiety disorders among siblings of subjects with SM and matched controls

Category	Subjects (*N* = 658) *n* (%)	Controls (*N* = 2092) *n* (%)	Model 1 Unadjusted OR (95% CI)	Model 2^a^ Adjusted OR (95% CI)	Model 3^b^ Adjusted OR (95% CI)
Generalized anxiety disorder	20 (3.0)	17 (0.8)	2.8 (1.5–5.4)*	3.4 (1.6–7.4)*	3.9 (1.6–9.7)*
Panic disorder and/or agoraphobia	19 (2.9)	30 (1.4)	1.6 (0.9–2.7)	N/A	N/A
Separation anxiety disorder	3 (0.5)	3 (0.1)	2.4 (0.5–12.1)	N/A	N/A
Social phobia	31 (4.7)	19 (0.9)	4.1 (2.3–7.7)***	3.3 (1.7–6.2)**	3.2 (1.5–6.7)*
Specific phobia	13 (2.0)	19 (0.9)	1.7 (0.8–3.3)	0.9 (0.4–2.3)	1.01 (0.3–3.4)
Unspecific anxiety disorders	82 (12.5)	99 (4.7)	2.1 (1.6–2.8)***	1.9 (1.4–2.6)**	1.7 (1.2–2.5)*
Selective mutism	48 (7.3)	3 (0.1)	42.2 (12.9–137.5)***	26.0 (8.1–83.0)***	27.9 (8.6–90.9)***

* < 0.05 **: < 0.001 ***: < 0.0001

^a^Model 2: adjusted for maternal mental disorder history, maternal socioeconomic status and marital status

^b^Model 3 (final model): adjusted for all covariates used in Model I and comorbidities of the subject with SM

The results of the additional sensitivity analyses for the subjects with SM born from 1995 to 2009 are in Supplementary Table 4. All diagnostic categories that were significant in the original data remained significant in the unadjusted analyses for children with SM born from 1995 to 2009.

The results of the sensitivity analyses for the subgroup of siblings of the subjects with SM that had not been diagnosed with anxiety disorders or childhood emotional disorders are presented in Supplementary Table 5. These show that all the same diagnostic groups, with the exception of affective disorders, remained significant in the unadjusted analyses when they were compared to the results for the complete data. The only group that lost significance, even though it was close to statistical significance, was affective disorders (OR 1.4, 95% CI 0.98–2.1, *P* = 0.0620), which included bipolar disorders and unipolar disorders. When unipolar disorders were analyzed as a separate group, the result was also statistically significant (*p* = 0.0386) for this subgroup.

The results from the drop-out analyses, which examined the subjects excluded due to ASD, psychotic disorders and moderate or severe mental disability, are presented in Supplementary Table 6. This table shows that there was no statistically significant difference between the number of mental disorder diagnoses given to the excluded subjects and the number given to the subjects who were included in the study (*P* = 0.0779). Moreover, the level of diagnosed childhood onset disorders did not differ between these two groups (*P* = 0.7994).

## Discussion

This nationwide population-based study examined mental disorder diagnoses among the siblings of children with SM, using a wide range of diagnoses, and a control group of children without SM and their siblings. There were two main findings. First, the siblings of children with SM had significantly more mental and neurodevelopmental disorders than the siblings of the controls. These associations were seen for a wide range of disorders and covariates only explained these findings to a small degree. The odds were highest for childhood emotional disorders and ASD when the results were adjusted for the maternal factors of SES, marital status and mental disorder diagnoses. Secondly, when various anxiety disorders were examined more closely, this showed that SM was more frequent among the siblings of children with SM than the siblings of the controls.

Our first main finding showed that the siblings of subjects with SM had a 1.5-fold increased risk for any mental or neurodevelopmental disorder and were two times more likely to have any childhood onset disorder than the siblings of the controls. The rate for any mental or neurodevelopmental disorder was as high as 41.2% in the siblings of children with SM, compared to 20.8% for the siblings of controls. Even though these percentages were much higher, they were still in line with the findings of a study by Kristensen et al. that reported that mental disorders were more common among the siblings of children with SM than the siblings of controls [13]. However, another study, based on interviews with parents, showed no increased association between SM and mental disorders in siblings [22]. This discrepancy may have been due to different research methods, as that study was based on a rather small sample.

When we examined specific disorder groups, the highest ORs were observed for childhood emotional disorders, ASD, ADHD and conduct disorders, which all are usually diagnosed during childhood. Our finding showed a higher level of childhood emotional disorders among the siblings of SM subjects and this was in line with previous studies [5, 23]. These studies reported that childhood emotional disorders, such as SM and childhood social phobias, occurred frequently among the siblings of subjects with SM [5, 23]. To the best of our knowledge, no previous studies have reported the prevalence of ADHD among the siblings of subjects with SM. Oppositional disorders have previously been associated with SM [23], but the findings were not uniform and it is unlikely that oppositional behavior plays an important role in the etiology of SM [5]. Our finding of the association between children with SM and siblings with ASD is particularly interesting, as associations between these two disorders have already been discussed in some studies [29, 30, 42]. These studies reported similarities in the communication features of both SM and ASD [29, 42] and that even some SM subjects could fulfill the diagnostic criteria for ASD [30]. However, no previous studies have examined ASD among the siblings of subjects with SM.

Our second main finding showed that when the various anxiety disorders were examined separately among the siblings, we found that the risk was also elevated for generalized anxiety disorders and non-specific anxiety disorders and not just for SM and social phobia. To the best of our knowledge, there have not been any previous findings of generalized anxiety disorders or non-specific anxiety disorders among siblings of subjects with SM. A meta-analysis of SM and anxiety disorder studies found that several comorbid anxiety disorders were also evident in subjects with SM [24]. This was supported by our finding that the association between SM and anxiety disorders was not just restricted to social phobia.

A previous study found that 21% of the siblings of subjects with SM had a history of social phobia and 19% had a history of SM [23]. Our findings also showed elevated levels of both disorders among the siblings of subjects with SM. A follow-up study on SM also found that 18% of the siblings had a history of mutistic reactions [7]. It is important to note this clustering of SM among siblings, because this same follow-up study on SM found that mutism in the core family could predict poor outcomes [7, 43]. Therefore, we need to pay particular attention to the treatment and follow-up of families when several children have SM. Different kinds of anxiety disorders have tended to co-aggregate among siblings [44] and this was confirmed by our findings that SM was associated with several different kinds of anxiety disorders in siblings.

To the best of our knowledge, this was the first study to investigate a wide range of diagnoses among the siblings of children with SM. Our findings, which showed a rather non-specific pattern of diagnoses among siblings, are novel. These findings also agree with the results of previous studies that showed shared genetic backgrounds and co-aggregation among mental disorder diagnoses [20, 45, 46]. Environmental factors could partly determine what kind of disorder a given subject develops. Non-shared environmental factors could partly explain why, in many cases, siblings developed different disorders to children with SM.

There could be other reasons for the strong association between SM and childhood onset disorders among siblings, in addition to shared genetic and environmental risk factors. It is possible that having one child with a mental disorder diagnosis in the family might make it easier for parents to notice different kinds of psychiatric symptoms and seek medical advice. Other risk factor for mental disorders could be sibling relationships and parents displaying different attitudes and educational styles toward siblings [47]. This is particularly likely when families already have one child with mental disorders. Previous studies have reported that the prevalence of ADHD in the general population was an average of 5% and varied from 2 to 7% [48]. The present study found that 5.8% of the siblings of children with SM had ADHD diagnoses and the rate was 2.0% for the siblings of the controls. Differences between help seeking by the families of the subjects and controls may partly explain this finding on ADHD. Adjusting for the comorbidities of the children with SM weakened the associations with conduct and oppositional disorders among their siblings, but the finding was still statistically significant in the final model.

Higher levels of autism-related symptoms have been observed by some studies on SM [30, 42] and it has been suggested that neurodevelopmental factors may play a role in the development of SM [26]. Further studies are needed to find out whether these findings were due SM and ASD having a shared etiological background. Alternatively, these could be explained, for example, by shared temperamental traits and tendencies to react by displaying behavioral inhibition [29]. It should be noted that ASD is an exclusion criterion for SM in ICD-10 and DSM-5 and that ASD causes communication problems and even global mutism. Despite this, our findings indicated the existence of family clustering of these two disorders and this should be investigated in more detail. Elevated levels of ASD and other mental and neurodevelopmental disorders have been observed among the siblings of subjects with ASD [18]. Having a child with ASD may cause family stress and create an environmental risk for SM [49], although our findings cannot be interpreted as causality.

The major strength of the present study was that we examined a large nationwide population-based sample. Register-based data made it possible to investigate a wide range of diagnoses and control the results by considering several covariates in the analyses. Unlike clinical studies, nationwide health registers are less likely to suffer from selection bias or sample loss at follow-up. The quality and accuracy of Finnish national registers has varied from satisfactory to very good, depending on the diagnosis [50]. The validity of SM diagnoses in a local subsample showed that as many as 87% of the subjects were diagnosed correctly [11]. The validity of ADHD, ASD and Tourette’s syndrome diagnoses has been shown to be very good [51–53]. Public healthcare is widely used in Finland and child healthcare is free of charge. The majority (99.5%) of children attend the free, routine health check-ups provided by primary care doctors and nurses, even if their family mainly uses private health care [33].

Despite the strengths of the study, there are some limitations that need to be considered. First, as the current study used register data, it was not possible to provide information on how the individual diagnoses were delivered or what diagnostic methods each clinic used. However, diagnoses used in the study were delivered by specialized health services and the validity of the Finnish registers have been shown to be good [50].

Second, the diagnostic methods and standards for registering diagnoses can change during long observation periods. To avoid any bias caused by changes in diagnostic classifications, we started observing sibling diagnoses from 1996, which was when Finland started using ICD-10. The mean age of the siblings at the end of the observation period was 15.6 ± 6.3 years and only 16 (0.3%) of the 5781 siblings were diagnosed when ICD-9 was in use. In addition, we controlled for changes in diagnostic methods over time, by conducting supplementary analyses of subjects born 1995–2009. These results did not differ from the results for the complete data.

Another limitation, which is specific to register-based case–control studies like the present one [54–58], is that some disorders that were examined were excluded from the controls. In this study, individuals with anxiety disorders and childhood onset emotional disorders were excluded from the controls. The excluded diagnoses did not include depressive disorders, as childhood depression is usually registered with an F32 diagnosis code. These exclusions ensured that the controls did not have an SM diagnosis or any of the disorders that were studied with the current data. However, this exclusion may also have affected how comparable the subjects and controls were to each other and how representative these results were of the general Finnish population. The cumulative incidence of anxiety disorders diagnosed by specialized health care was 5.7% from 1992 to 1996 [59], so it is unlikely that excluding controls with these diagnoses would have explained the results completely. When we used sensitivity analyses to compare the siblings of subjects with SM that did not have comorbid anxiety disorders or childhood onset emotional disorders to the siblings of the controls, most of the same diagnostic group differences remained significant. Therefore, it is unlikely that the results can be explained by a lack of anxiety disorders and childhood onset emotional disorders among the controls. However, excluding anxiety disorders and childhood onset emotional disorders might have affected the results of some of the individual diagnostic groups, namely anxiety disorders, and further studies with more representative data are warranted.

Finally, as the data often included more than one sibling for each subject, multilevel modeling could have been the appropriate way to deal with the variations among the families. Unfortunately, there were not enough dimensions of variation in the present data set to use this method. The marginal model was the most appropriate study design, because our main interest was to compare mental disorders between the siblings of the subjects and the siblings of the controls, rather than between the families in the two groups.

## Conclusions

This study found elevated levels of mental and neurodevelopmental disorders among the siblings of children with SM. The associations were stronger for childhood onset disorders, which may indicate shared genetic and environmental risk factors. The association with ASD in the siblings of children with SM is particularly interesting, because of previously reported associations between these two disorders. Family studies that focus on both SM and ASD are needed, as well as further research on the shared etiologies of other anxiety disorders and SM. It is important for clinicians to identify the siblings of children with SM at an early stage, as they have a higher risk of developing mental disorders than the general population.

### Supplementary Information

Below is the link to the electronic supplementary material.

Supplementary file1 (DOCX 42 KB)

## Data Availability

The data in this paper were based on Finnish national health registers and cannot be shared online. All the material and tables in this paper are available in the paper or as supplementary tables.
