# Supplementary material for: Selective mutism and the risk of mental and neurodevelopmental disorders among siblings
Source: Eur Child Adolesc Psychiatry. 2022 Nov 23;33(1):291–302. doi: 10.1007/s00787-022-02114-3 (PMC10805856; doi:10.1007/s00787-022-02114-3)
Supplement: Supplementary file 1 — Supplementary file1 (DOCX 42 KB) [file 787_2022_2114_MOESM1_ESM.docx]

**Supplementary information**

# Selective mutism and the risk of mental and neurodevelopmental disorders among siblings

European Child and Adolescent Psychiatry

Miina Koskela1,2,3, Elina Jokiranta-Olkoniemi1,2,3,4, Terhi Luntamo1,2, Auli Suominen1,2,3, Andre Sourander1,2,3**, Hans-Christoph Steinhausen5,6,7,8**

1. Department of Child Psychiatry, University of Turku, Finland.
2. Department of Child Psychiatry, Turku University Hospital, Finland.
3. INVEST Research Flagship Center, University of Turku, Turku, Finland.
4. Unit of Psychology, Faculty of Education, University of Oulu, Oulu, Finland
5. Department of Child and Adolescent Psychiatry, Psychiatric University Clinic, Zurich, Switzerland
6. Clinical Psychology and Epidemiology, Department of Psychology, University of Basel, Switzerland
7. Department of Child and Adolescent Psychiatry, University of Southern Denmark, Odense, Denmark
8. Child and Adolescent Mental Health Centre, Capital Region Psychiatry, Copenhagen, Denmark.

**Joint senior authors

**Corresponding author:**

Miina Koskela

Email: miikosy@utu.fi

Supplementary Table 1: Diagnostic categories for mental diagnoses in the siblings

|  | **ICD-10** | **ICD-9** |
| --- | --- | --- |
| **1.Any mental or neurodevelopmental disorder** | F10-F99 | 291-319, excluding 316 |
| a. Schizophrenia spectrum disorders | F20 schizophrenia, F21  schizotypal disorder, F22  delusional disorder, F23 acute  polymorphic psychotic disorder without symptoms of  schizophrenia, F24 induced  delusional disorder, F25  schizoaffective disorder, F28  other nonorganic psychotic  disorders, F29 unspecified  nonorganic disorders | 295, 297, 2989X, 3012C |
| b. Affective disorders | F30 hypomania, F31 bipolar  affective disorder, F32  depressive episode, F33  recurrent depressive episode,  F34 cyclothymia and dysthymia,  F38 other single mood  (affective) disorder, F39  unspecified mood (affective)  disorder | 296, 3004A, 2988A |
| *Bipolar disorders* | *F30, F31* | *2962A-G, 2963A-G, 2964A-G, 2967A* |
| *Unipolar disorders* | *F32, F33, F34, F38, F39* | *2961A-G, 2968A, 3004A, 2988A* |
| c. Anxiety disorders | F40 phobic anxiety disorders,  F41 other anxiety disorders  (excluding F41.2), F42  obsessive– compulsive disorder | 3000A, 3000B, 3000C, 3002B,  3002C, 3002D, 3002X, 3003A |
| d. Other neurotic and personality disorders | F41.2 mixed anxiety and  depression, F43 reaction to  severe stress and adjustment  disorders, F44 dissociative  amnesia, F45 somatoform  disorders, F48 other neurotic  disorders, F50 eating disorders,  F51 non-organic sleep disorders  (excluding F51.3; F51.4), F52  lack or loss of sexual desire,  F53 mental and behavioral  disorders associated with the  puerperium, not elsewhere  classified, F54 psychological  and behavioral factors  associated with disorders or  diseases classified elsewhere,  F55 abuse of non-dependence  producing substances, F59  unspecified behavioral  syndromes associated with  physiological disturbances, F60  specific personality disorders,  F61 mixed and other personality disorders, F62 enduring  personality changes, not  attributable to brain damage and diseases, F63 habit and impulse disorders, F64 gender identity disorders, F65 fetishism, F66 psychological and behavioural disorders associated with sexual  development and orientation,  F68 other disorders of adult  personality and behavior, F69  unspecified disorder of adult  personality and behavior, F99  mental disorder not otherwise  specified | 300–302 (excluding 3000A,  3000B, 3000C, 3002B, 3002C,  3002D, 3002X, 3003A, 3004A and  3012C), 3071A, 3074A, 3074F,  3074H, 3075A, 3075B, 3075C,  3075E, 3078A, 3079X, 309  (excluding 3092A and 3092B), 312 (excluding 3120A, 3123C and  3123D) |
| e. Substance abuse disorder | Mental and behavioural  disorders due to use of… F10  alcohol, F11 opioids, F12  cannabinoids, F13 sedatives or  hypnotics, F14 cocaine, F15  other stimulants, including  caffeine, F16 hallucinogens,  F17 tobacco, F18 volatile  solvents, F19 multiple drug use  and use of other psychoactive  substances | 303-305, 291-292 |
| **2. Childhood onset disorders** |  |  |
| a. Autism spectrum disorders | F84 | 299 |
| b. Attention deficit hyperactivity disorder | F90 hyperkinetic disorders | 314 |
| c. Intellectual disability | F70-F79 intellectual disability | 317-319 |
| d. Childhood emotional disorders | F93, F94 emotional disorders with  onset specific to childhood | 3092A, 3092B, 3133A, 3132C, 3138C |
| e. Conduct and oppositional disorders | F91-F92 conduct disorders,  including oppositional defiant  disorder | 3120A, 3123C, 3123D, 3138A |
| f. Tic disorders | F95 tic disorders | 3072A, 3072B, 3072C, 3072D |
| g. Learning and coordination disorders | F80-F83 learning disabilities or  motor coordination disorders,  including developmental  disorders of speech, language,  scholastic skills or motor  coordination | 315 |

Supplementary Table 2: Diagnostic categories for various anxiety disorders

| **Diagnostic category** | **ICD-10** |
| --- | --- |
| Generalized anxiety disorder | F41.1, F93.80 |
| Panic disorder and/or agoraphobia | F40.00, F40.01, F41.00, F41.01, F41.08, F41.09 |
| Separation anxiety disorder | F93.0 |
| Social phobia | F40.1, F93.2 |
| Specific phobia | F40.2, F93.1 |
| Unspecific anxiety disorders | F40.8, F40.9, F41.2, F41.3, F41.8, F41.9, F93.89, F93.9 |
| Selective mutism | F94.0 |

Supplementary Table 3: Covariate testing for mental disorders in siblings of children with SM and controls (N=436/20.8%)

|  | Sibling mental disorders | | | Relation between covariates and outcome |
| --- | --- | --- | --- | --- |
| Covariates |  |  | **P-value (**χ2) | **P-value (**χ2) |
|  | **Yes** | **No** |  |  |
| Maternal socioeconomic status^a^ |  |  | 0.0542 | <0.0001 |
| Upper white-collar worker | 53 (14.6) | 283 (20.1) |  |  |
| Lower white-collar worker | 158 (43.4) | 615 (43.7) |  |  |
| Blue-collar worker | 71 (19.5) | 246 (17.5) |  |  |
| Other | 82 (22.5) | 262 (18.6) |  |  |
| Maternal marital status^b^ |  |  | 0.0909 | 0.0167 |
| Married/cohabitating | 389 (97.0) | 1520 (98.3) |  |  |
| Single | 12 (3.0) | 26 (1.7) |  |  |
| Paternal mental disorders |  |  | 0.0558 | 0.0001 |
| Range | 0-29 |  |  |  |
| Mean (SD) | 0.4 (1.6) |  |  |  |
| Maternal mental disorders |  |  | 0.0018 | <0.0001 |
| Range | 0-38 |  |  |  |
| Mean (SD) | 0.4 (1.8) |  |  |  |
| Maternal age |  |  | 0.4324 | 0.4550 |
| Range | 16.0-46.0 |  |  |  |
| Mean (SD) | 29.1 (5.0) |  |  |  |
| Paternal age |  |  | 0.2178 | 0.0024 |
| Range | 16.0-59.0 |  |  |  |
| Mean (SD) | 31.5 (5.8) |  |  |  |

^a^ Missing information on maternal socioeconomic status: 94 subjects (14.3%) and 322 controls (15.4%)

^b^Missing information on maternal marital status: 37 subjects (5.6%) and 145 controls (6.9%)

Supplementary Table 4: Additional sensitivity analyses for the group of subjects with SM born in 1995-2009

| Sibling diagnosis | All subjects (N=658)  n (%) | All controls (N=2092)  n (%) | Unadjusted OR  (95% CI) | P-value | Subjects born 1995-2009  (N= 474)  n (%) | Controls born 1995-2009 (N=1526)  n (%) | Unadjusted OR  (95% CI) | P-value |
| --- | --- | --- | --- | --- | --- | --- | --- | --- |
| Any mental or neurodevelopmental disorder | 271 (41.2) | 435 (20.8) | 1.7 (1.5-1.9) | **<0.0001** | 197 (41.6) | 299 (19.6) | 1.9 (1.6-2.3) | **<0.0001** |
| Schizophrenia spectrum disorders | 16 (2.4) | 25 (1.2) | 1.5 (0.8-2.7) | 0.2367 | 8 (1.7) | 15 (1.0) | 1.0 (0.4-3.1) | 0.9447 |
| Affective disorders | 100 (15.2) | 165 (7.9) | 1.5 (1.2-1.9) | **0.0006** | 60 (12.7) | 91 (6.0) | 2.0 (1.4-2.9) | **<0.0001** |
| *Bipolar disorders* | 2 (0.3) | 16 (0.8) | 0.3 (0.1-1.3) | 0.0979 | 2 (0.4) | 7 (0.5) | 0.7 (0.1-3.5) | 0.6750 |
| *Unipolar disorders* | 99 (15.1) | 158 (7.6) | 1.6 (1.2-2.0) | **0.0003** | 59 (12.5) | 87 (5.7) | 2.1 (1.5-3.0) | **<0.0001** |
| Anxiety disorders | 76 (11.6) | 110 (5.3) | 1.8 (1.4-2.5) | **<0.0001** | 50 (10.6) | 67 (4.4) | 2.1 (1.4-3.2) | **0.0003** |
| Other neurotic and personality disorders | 74 (11.3) | 159 (7.6) | 1.2 (0.9-1.5) | 0.2370 | 51 (10.8) | 94 (6.2) | 1.4 (0.98-2.1) | 0.0612 |
| Substance abuse disorders | 18 (2.7) | 42 (2.0) | 1.1 (0.7-1.8) | 0.7278 | 7 (1.5) | 23 (1.5) | 0.8 (0.3-2.0) | 0.6924 |
| Any childhood onset disorder | 198 (30.1) | 222 (10.6) | 2.3 (1.9-2.8) | **<0.0001** | 151 (31.9) | 175 (11.5) | 2.4 (1.9-3.0) | **<0.0001** |
| Autism spectrum disorders | 23 (3.5) | 20 (1.0) | 2.6 (1.4-4.8) | **0.0016** | 19 (4.0) | 15 (1.0) | 3.7 (1.8-8.0) | **0.0007** |
| Attention deficit hyperactivity disorder | 38 (5.8) | 41 (2.0) | 2.3 (1.5-3.5) | **0.0002** | 30 (6.3) | 35 (2.3) | 2.3 (1.4-3.9) | **0.0015** |
| Intellectual disability | 12 (1.8) | 19 (0.9) | 1.6 (0.8-3.5) | 0.1921 | 8 (1.7) | 15 (1.0) | 1.5 (0.6-3.8) | 0.3882 |
| Childhood emotional disorders | 95 (14.4) | 39 (1.9) | 6.1 (4.2-8.9) | **<0.0001** | 76 (16.0) | 33 (2.2) | 6.9 (4.4-10.8) | **<0.0001** |
| Conduct and oppositional disorder | 35 (5.3) | 40 (1.9) | 2.2 (1.4-3.5) | **0.0009** | 27 (5.7) | 27 (1.8) | 3.0 (1.7-5.5) | **0.0003** |
| Tic disorders | 7 (1.1) | 14 (0.7) | 1.4 (0.5-3.6) | 0.4874 | 5 (1.1) | 11 (0.7) | 1.2 (0.4-4.1) | 0.7234 |
| Learning and coordination disorders | 104 (15.8) | 136 (6.5) | 1.9 (1.5-2.4) | **<0.0001** | 79 (16.7) | 107 (7.0) | 2.0 (1.5-2.7) | **<0.0001** |

Supplementary Table 5: Additional sensitivity analyses for the group of subjects with SM without anxiety disorders of childhood onset disorders

| Sibling diagnosis | All subjects (N=658)  n (%) | All controls (N=2092)  n (%) | Unadjusted OR  (95% CI) | P-value | Subjects, without ANX*  (N= 447)  n (%) | Controls,  without ANX*  (N=1426)  n (%) | Unadjusted OR  (95% CI) | P-value |
| --- | --- | --- | --- | --- | --- | --- | --- | --- |
| Any mental or neurodevelopmental disorder | 271 (41.2) | 435 (20.8) | 1.7 (1.5-1.9) | **<0.0001** | 175 (39.2) | 284 (19.9) | 1.7 (1.4-2.1) | **<0.0001** |
| Schizophrenia spectrum disorders | 16 (2.4) | 25 (1.2) | 1.5 (0.8-2.7) | 0.2367 | 8 (1.8) | 14 (1.0) | 1.5 (0.6-3.8) | 0.3635 |
| Affective disorders | 100 (15.2) | 165 (7.9) | 1.5 (1.2-1.9) | **0.0006** | 54 (12.1) | 104 (7.3) | 1.4 (0.98-2.1) | 0.0620 |
| *Bipolar disorders* | 2 (0.3) | 16 (0.8) | 0.3 (0.1-1.3) | 0.0979 | 1 (0.2) | 8 (0.6) | 0.4 (0.04-2.9) | 0.3359 |
| *Unipolar disorders* | 99 (15.1) | 158 (7.6) | 1.6 (1.2-2.0) | **0.0003** | 54 (12.1) | 101 (7.1) | 1.5 (1.02-2.2) | **0.0386** |
| Anxiety disorders | 76 (11.6) | 110 (5.3) | 1.8 (1.4-2.5) | **<0.0001** | 43 (9.6) | 64 (4.5) | 1.7 (1.1-2.7) | **0.0151** |
| Other neurotic and personality disorders | 74 (11.3) | 159 (7.6) | 1.2 (0.9-1.5) | 0.2370 | 40 (8.9) | 99 (6.9) | 1.1 (0.8-1.7) | 0.5007 |
| Substance abuse disorders | 18 (2.7) | 42 (2.0) | 1.1 (0.7-1.8) | 0.7278 | 8 (1.8) | 28 (2.0) | 0.7 (0.3-1.6) | 0.3713 |
| Any childhood onset disorder | 198 (30.1) | 222 (10.6) | 2.3 (1.9-2.8) | **<0.0001** | 134 (30.0) | 146 (10.2) | 2.3 (1.8-3.0) | **<0.0001** |
| Autism spectrum disorders | 23 (3.5) | 20 (1.0) | 2.6 (1.4-4.8) | **0.0016** | 14 (3.3) | 11 (0.8) | 4.1 (1.7-9.6) | **0.0013** |
| Attention deficit hyperactivity disorder | 38 (5.8) | 41 (2.0) | 2.3 (1.5-3.5) | **0.0002** | 25 (5.6) | 23 (1.6) | 2.9 (1.6-5.4) | **0.0006** |
| Intellectual disability | 12 (1.8) | 19 (0.9) | 1.6 (0.8-3.5) | 0.1921 | 11 (2.5) | 12 (0.8) | 2.3 (0.9-6.0) | 0.0941 |
| Childhood emotional disorders | 95 (14.4) | 39 (1.9) | 6.1 (4.2-8.9) | **<0.0001** | 64 (14.3) | 23 (1.6) | 6.3 (3.7-10.5) | **<0.0001** |
| Conduct and oppositional disorder | 35 (5.3) | 40 (1.9) | 2.2 (1.4-3.5) | **0.0009** | 16 (3.6) | 26 (1.8) | 2.3 (1.1-4.9) | **0.0359** |
| Tic disorders | 7 (1.1) | 14 (0.7) | 1.4 (0.5-3.6) | 0.4874 | 2 (0.5) | 9 (0.6) | 0.6 (0.1-3.0) | 0.5507 |
| Learning and coordination disorders | 104 (15.8) | 136 (6.5) | 1.9 (1.5-2.4) | **<0.0001** | 78 (17.5) | 99 (6.9) | 2.1 (1.6-2.8) | **<0.0001** |

*ANX= anxiety disorders of childhood onset disorders

Supplementary Table 6: descriptive information on the excluded subjects

|  | Excluded subject  N= 243 | Subjects in complete data N= 658 | P-value |
| --- | --- | --- | --- |
| **Sibling mental disorders** |  |  |  |
| *Any mental or neurodevelopmental disorder* | 116 (47.7) | 271 (41.2) | 0.0779 |
| *Any childhood onset disorder* | 71 (29.2) | 198 (30.1) | 0.7994 |
| **Maternal SES*** |  |  | 0.5749 |
| *Upper white-collar worker* | 22 (11.4) | 54 (9.6) |  |
| *Lower white-collar worker* | 80 (41.5) | 229 (40.6) |  |
| *Blue-collar worker* | 43 (22.3) | 152 (27.0) |  |
| *Other* | 48 (24.9) | 129 (22.9) |  |

* Missing information on maternal socioeconomic status. In excluded data: 50 subjects (20.6%). In complete data: 94 subjects (14.3%)
